# Bruns Garland Syndrome as the first presentation of type 2 diabetes: two case reports and a practical approach to diagnosis

**DOI:** 10.1186/s13256-023-04327-9

**Published:** 2024-02-16

**Authors:** Sathyajith Ambawatte, Piyumi Wijewickrama, Kamal Gunarathne, Noel Somasundaram

**Affiliations:** 1 Ministry of Health Sri Lanka, Colombo, Sri Lanka; 2 Neuro-Electrophysiology Unit, National Hospital of Sri Lanka, Colombo, Sri Lanka; 3 Diabetes & Endocrinology, Unit National Hospital of Sri Lanka, Colombo, Sri Lanka

**Keywords:** Diabetes, Type 2 diabetes, Diabetic neuropathy, Bruns–Garland syndrome, Diabetic radiculoplexus neuropathy

## Abstract

**Background:**

Diabetes is a global health problem causing a significant burden on the healthcare systems both due to the disease itself and associated complications. Diabetic radiculoplexus neuropathies or Bruns–Garland syndrome constitutes a rare form of microvascular complications, more commonly affecting the lumbosacral plexus and, very rarely, the cervical plexus. We describe two Sri Lankan males who presented with diabetic lumbosacral radiculoplexus neuropathy and diabetic cervical radiculoplexus neuropathy as the initial manifestation of diabetes.

**Case description:**

Case 1: a 49-year-old Sri Lankan hotel chef presented with subacute painful weakness and wasting of the left upper arm for 3 months and weight loss. Left upper limb proximal muscles were wasted with diminished power and reflexes. A nerve conduction study showed comparative amplitude reduction. An electromyogram revealed positive sharp waves, frequent fibrillations, and high amplitude polyphasic motor unit potentials with reduced recruitment in proximal muscles of left upper limb. Case-2: a 47-year-old Sri Lankan carpenter presented with subacute progressive asymmetrical painful weakness and wasting of bilateral thighs for 5 months and weight loss. Lower limb proximal muscles were wasted with reduced power and knee jerks. The nerve conduction study was normal. The electromyogram was similar to case 1 involving both quadratus femoris muscles, which was more prominent on the left side. The work up for an underlying etiology revealed only elevated fasting blood glucose and HbA1c, suggesting a new diagnosis of diabetes associated with neurological symptoms. Patient 1 was diagnosed with diabetic cervical radiculoplexus neuropathy and patient 2 with diabetic lumbosacral radiculoplexus neuropathy. Both showed significant improvement following optimization of glycemic control together with symptomatic treatment and physiotherapy.

**Conclusion:**

Diagnosis of diabetic radiculoplexus neuropathy requires a comprehensive workup to rule out other sinister pathologies. This case report has a dual importance; it describes diabetic radiculoplexus neuropathy as the very first manifestation of two previously healthy people, giving rise to a new diagnosis of diabetes and, at the same time, reporting on diabetic cervical radiculoplexus neuropathy, which is extremely rare and has never been previously reported in Sri Lanka.

## Background

Diabetes is a global health concern with a significant impact on the people, society, and the economy of a country. Data from the International Diabetes Federation diabetes atlas reveal that approximately 463 million adults were suffering from diabetes by 2019, and this number is projected to increase up to 578 million by 2030 [1]. Out of all the south Asian countries, Sri Lanka has the second highest age adjusted comparative diabetes prevalence among 20–79 years age, at 10.7% [1].

Out of the diabetes-related neuropathies, peripheral neuropathy is the most common, with a prevalence of 16–57% among adults, while diabetes-related painful neuropathies affect 26% of adults [2, 3].

Diabetes neuropathies are broadly classified as diffused neuropathies, mononeuropathy and radiculopathy or polyradiculopathy [4]. Diabetic polyradiculopathy is a rare entity associated with significant pain and disability. This is also known as diabetic amyotrophy, Bruns–Garland syndrome, proximal diabetic neuropathy, diabetic myelopathy, diabetic motor neuropathy, diabetic radiculoplexopathy and diabetic radiculoplexus neuropathies (DRPN) [5]. This is frequently referred to as diabetic lumbosacral radiculoplexes neuropathy (DLRPN), indicating the more common involvement of lumbosacral roots, which affects lower limbs [5]. The presence of a variety of terms for this disease entity implies the variable nature of opinions on pathophysiology and the extent of involvement in this condition.

DRPN as the first presentation of diabetes is rare and has never been reported in Sri Lanka. DRPN affecting the cervical radiculoplexus is rarer. We describe two cases with DLRPN and diabetic cervical radiculoplexus neuropathy (DCRPN) as the first presentation of type 2 diabetes in Sri Lanka.

## Case presentation

### Patient 1

A 49-year-old, previously healthy Sri Lankan hotel chef presented with subacute painful weakness and reduced muscle bulk of the left upper arm for 3 months, without contralateral upper limb or lower limb involvement. He did not have neck pain, history of trauma or associated autonomic symptoms. He had a significant weight loss of 7 kg over last 3 months, without a change in appetite. There were no symptoms or signs of other systemic involvement. He was clinically euthyroid. He did not have a history of diabetes or hyperglycemic symptoms including polyuria or polydipsia. There was no history to suggest an ongoing vasculitis or a connective tissue disorder.

He had no family history of similar neurological disorders, diabetes, or malignancies. The disability due to his ongoing symptoms involving the upper arm was having a significant effect on his occupation as a hotel chef.

**Fig. 1:**
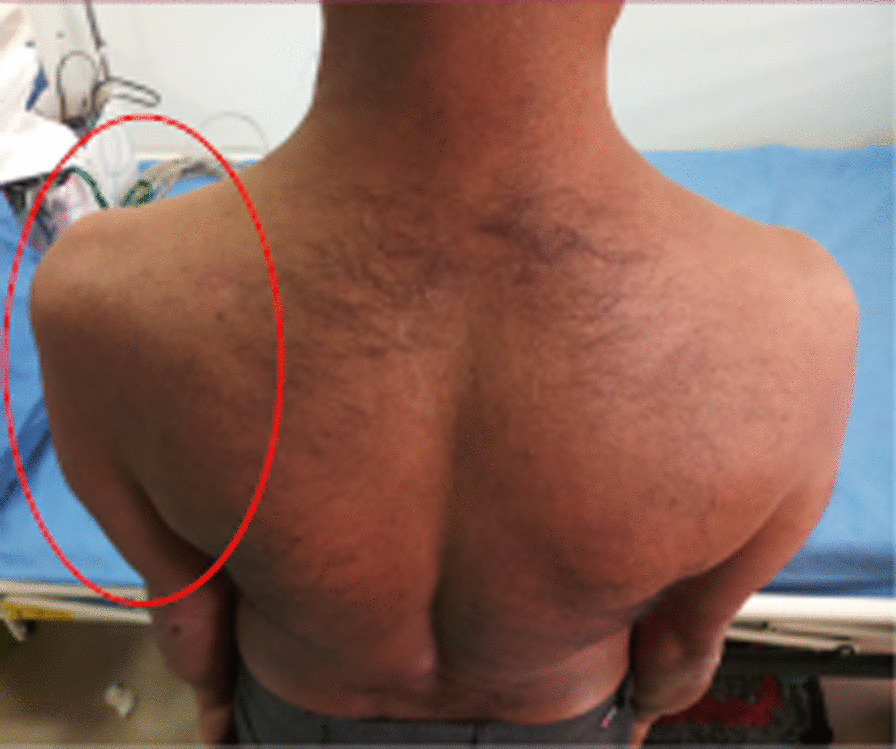
Wasting of left supraspinatus, deltoid, infraspinatus, teres major, and triceps (published with the patient’s consent)

He was thin built with a Body Mass Index (BMI) of 19 kg/m^2^ without any features of insulin resistance. Neurological examination of his left upper limb revealed muscle wasting of shoulder girdle and biceps (Fig. 1) without fasciculations, and proximal muscle strength was reduced to 4/5 of the Medical Research Council (MRC) muscle strength scale, with diminished bicep and triceps jerks. Sensory examination was normal. Right upper limb, lower limbs, cranial nerves, fundi, and higher function examinations were normal.

His systemic examination was unremarkable.

His initial investigations revealed a high erythrocyte sedimentation rate (ESR) at 60 mm/hour, with normal C-reactive protein (CRP). His cerebrospinal fluid (CSF) examination showed albuminocytological dissociation with high CSF protein at 70 mg/dl and an unremarkable high volume cytospin.

His magnetic resonance imaging (MRI) of the cervical and thoracic spine with brachial plexus was normal. The only other significant investigation finding was high fasting plasma glucose (FPG) at 198 mg/dl and HbA1c of 9%, confirming a new diagnosis of diabetes. His C-peptides were within normal range, with a negative type 1 diabetes antibody panel. He had a negative vasculitis screening and no evidence of an ongoing malignancy.

Nerve conduction studies (NCS) revealed comparative amplitude reduction in left upper limb in both sensory and motor studies with normal conduction velocity and lower limb NCS. An electromyelogram (EMG) demonstrated positive sharp waves, frequent fibrillations, and high amplitude polyphasic motor unit potentials with reduced recruitment in proximal muscles of left upper limb, suggestive of a neurogenic process.

The final diagnosis was diabetes cervical radiculoplexus neuropathy as the first presentation of type 2 diabetes, without other associated microvascular or macrovascular complications. He was initiated on oral antidiabetic medications, which gradually improved his glycemic control. This was followed by improvement of his neurological impairment over a few months, without any residual weakness. The patient was monitored for 3 years and showed no signs or symptoms of an alternative diagnosis or relapse.

### Patient 2

A 47-year-old, previously healthy Sri Lankan carpenter presented with subacute progressive asymmetrical painful weakness and wasting of both thighs for 5 months, affecting his functional capacity and limiting his mobility. There was no associated numbness or upper limb involvement. He also noted a weight loss of 5 kg over 1 month with worsening fatigue. The rest of his systemic inquiry was normal, without a significant past medical history or family history. He had been engaging in heavy work as a part of his occupation. However, there was no history of trauma to his back.

**Fig. 2:**
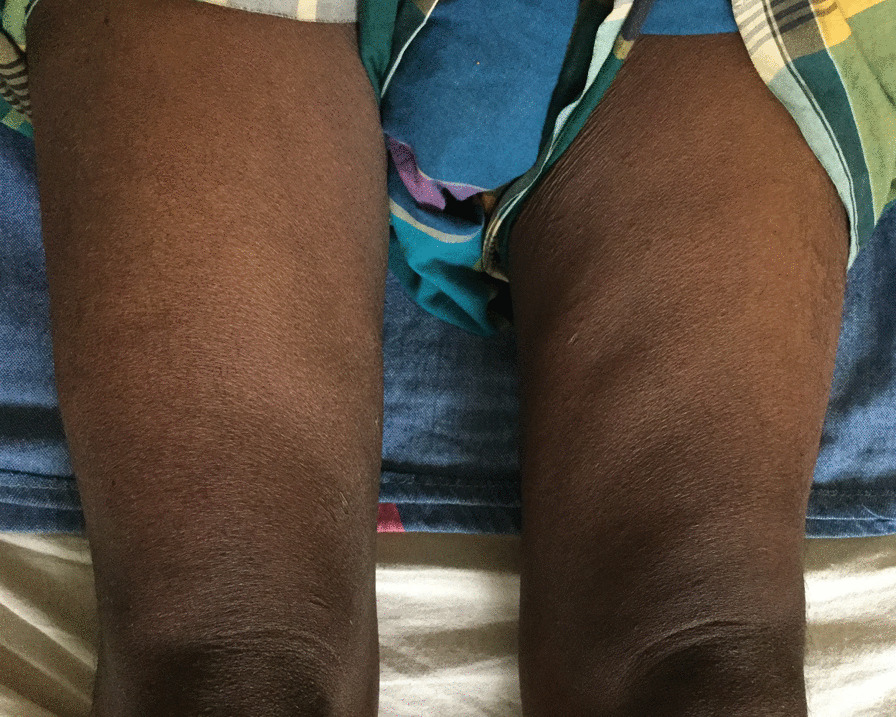
(Published with patient’s consent) Wasting of both quadriceps

He had a BMI of 21 kg/m^2^ and early acanthosis nigricans. A lower limb examination revealed proximal muscle wasting, more prominent on left than right (Fig. 2), without fasciculations. There was reduced power in proximal muscles, with MRC muscle strength of 3/5 on the left and 4/5 on the right, with diminished knee jerks. The rest of the lower limb examination, including sensory examination, was normal.

Similar to patient 1, patient 2’s ESR was 72 mm/hour with normal CRP, and CSF was acellular with an elevated protein level of 89 mg/dl and an unremarkable high volume cytospin. His MRI of the lumbosacral spine and lumbosacral plexus were normal, and extensive screening for metabolic, nutritional, oncological and autoimmune etiology was unremarkable.

Interestingly, his FPG was elevated at 210 mg/dl, with an HbA1C of 10.2%. An EMG revealed positive sharp waves, frequent fibrillations, and high amplitude polyphasic motor unit potentials with reduced recruitment in both quadratus femoris muscles; the findings were more prominent on left side, supporting an asymmetric neurogenic process. The NCS was unremarkable.

Therefore, this patient was diagnosed with lumbosacral radiculoplexus neuropathy presenting as the initial manifestation of type 2 diabetes mellitus. He was started on dual oral hypoglycaemic medications, which lead to an improvement of his neurological manifestations, including pain over a few months with optimum glycemic control and rehabilitation. The patient was followed for 3 years and did not have a relapse or symptoms or signs of an alternative diagnosis.

## Discussion

These two cases are unique for several reasons. Radiculoplexus neuropathies are a rare phenomenon seen in diabetes, affecting only 4.16 per 100,000 population [6]. Diabetes cervical radiculoplexus neuropathy, as seen in patient 1, is rare and can be misdiagnosed as plexopathy, cervical neuropathy, or a mononeuropathy, which are more common causes for weakness and wasting in this region.

Ludwig Bruns, a German neurologist, described three patients in 1890 who had significant discomfort in their hips and thighs, as well as weakening and wasting of leg muscles, but no objective sensory loss [7]. Following this publication, there were only a few incidents that could be described in the same way. Hugh Garland and Deryck Taverner described five individuals with the same condition in 1953 under the title “Diabetic Myelopathy,” which was followed by another paper by Hugh Garland titled “Diabetic Amyotrophy,” which contained seven new patients and follow-up of the original cases [8, 9].

In 2012, clinical, laboratory, neurophysiological, imaging, and pathological characteristics of 85 patients with diabetes presenting with similar upper-limb syndrome suggested that this condition could affect the upper limbs and was termed “diabetes cervical radiculoplexus neuropathy,” with the caveat that this syndrome could spread to the cranial and thoracic nerves as well [10]. Case 1, as mentioned above, describes this rare entity of DCRPN, which was, in fact, the presenting symptom of diabetes in this patient.

A study done in USA over a 16-year period revealed that DLRPN constitutes two-thirds of all lumbosacral radiculoplexes neuropathies (LRPN), while one-third are idiopathic. The incidence of LRPN in this population was 4.16 per 100,000 population per year [6].

DRPN usually affects males over the age of 50 with type 2 diabetes and can rarely affect women and younger people with diabetes [11]. This could develop as a consequence of a prediabetic state or strict glycemic control in someone who has just been diagnosed with diabetes [12]. The pain usually initiates in the thigh or buttock of one side, then extends to other regions of the same leg, followed by contralateral involvement. The pain is followed by weakness and, eventually, muscle atrophy and weight loss [13]. Usually, the proximal involvement is more common compared with the distal involvement. Spread to the contralateral limb will take a median time of 3 months and can be up to 72 months. A very rare sensory predominant clinical presentation is also described, but with neurophysiological studies showing predominant motor involvement. Uncommonly, patients may present with a painless form, which is common in upper limbs with an insidious onset, symmetric, and slowly progressive phenotype [7]. Apart from this, associated weight loss of more than 4.5 kg was described in some patients, as well as associated new autonomic symptoms being prevalent in 50% [5, 9].

DRPN could be triggered by tight glycemic control, initiation of hypoglycemic medications, immunization, trauma, or infection. In both patients described above, no such triggers were identified, and DRPN was indeed the initial presentation that lead to a new diagnosis of diabetes, which is an extremely rare occurrence.

The pathophysiology of DRPN is incompletely described. Specific changes in the nerve biopsy will be seen as focal or multifocal nerve fiber degeneration and epineural neovascularization (suggestive of vasculopathy), and inflammatory infiltrates in small arterioles, venules, and capillaries (suggestive of microvasculitis with or without necrosis), were observed in nerve biopsies [9]. Immunostaining revealed increased ICAM-1 positive cells and increased NF-κB staining in blood vessels. Both findings support an underlying dysimmune vascular basis [14]. Multiple post mortem studies have demonstrated immune mediated microvasculitis as the underlying mechanism of DRPN, rather than the chronic hyperglycaemia [5, 15, 16].

**Fig. 3:**
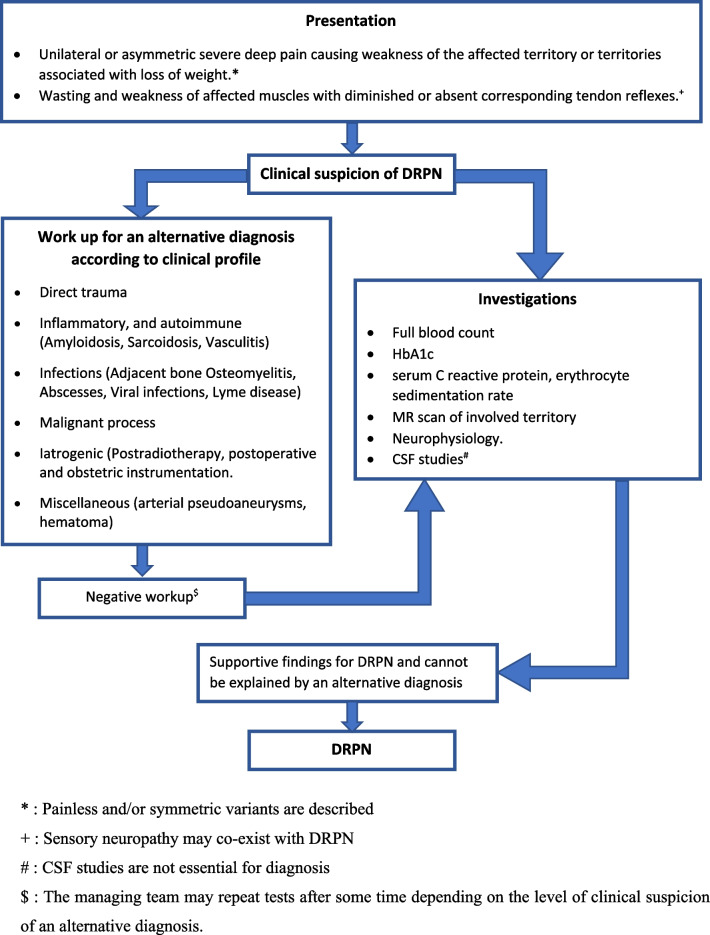
Algorithm for diagnosis of diabetes radiculoplexus neuropathy. *DRPN* diabetes radiculoplexus neuropathy, *MR* magnetic resonance, *CSF* cerebrospinal fluid

Vasculitis (primary or secondary), compressive plexopathy, and infiltrative causes, such as malignancies, sarcoidosis, amyloidosis, and abscess (tuberculosis and *Salmonella*), are all plausible etiologies for radiculoplexus neuropathies that should be looked for in any patient with a similar presentation [13]. Figure 3 summarizes the important alternative diagnoses. Repeated CSF studies, including high volume cytospin, extensive vasculitic screening, advanced imaging such as fluorodeoxyglucose (FDG)-positron emission tomography (PET), and nerve biopsy, may be required when there is a higher suspicion of an alternative diagnosis. The focused work-up plan for DRPN is summarized in Fig. 3

Nerve conduction studies are unreliable to demonstrate the involvement of plexus. Sensory amplitudes may be reduced, indicating postganglionic involvement, and compound muscle action potential amplitudes may be reduced, with a slight drop in conduction velocity indicating axonal loss [13]. Patient 1 had reduced amplitude on the affected side, and patient 2 had a normal NCS.

Electromyography is highly informative in this context since it indicates fibrillation potentials, extended duration, and high amplitude motor unit action potentials in the afflicted muscle groups, with numerous myotomes frequently involved [13], which were apparent in both patients.

While the inflammatory markers may be high, generally the ESR is known to be below 50 mm/hour [17]. Both of our patients exhibited an elevated ESR of more than 50 mm/hour with normal CRP. There is no evidence in the literature mentioning a dissociation between ESR and CRP in DRPN, as seen in both of our patients.

Nerve root enhancement on MRI with gadolinium of the afflicted nerve roots may be seen in a subset of patients with DRPN, and it suggests a higher degree of inflammatory burden, indicating the necessity of immunosupression [18]. However, both of our patients’ plexus MRIs were unremarkable, which may indicate that the inflammatory burden was not significant enough to show an enhancement.

On CSF examination, albuminocytologic dissociation is well characterized in DRPN and was observed in both of our cases. CSF examination is useful to establish inflammation. CSF evaluation with high volume cytospine is when there is suspicion of an infiltrative cause and can be repeated if necessary when there are ongoing concerns. A nerve biopsy is not indicated to achieve a diagnosis of DRPN but indeed is useful in the context of unusual presentations with a greater suspicion of an infiltrative etiology. Coupling nerve biopsy with muscle biopsy will improve the yield in patients with suspected vasculitis [19].

Alternative treatment modalities, including steroids, immunoglobulin, and plasma exchange, have been studied in a small number of patients with severe cases but inconclusive evidence [13]. Therefore, the mainstay of treatment remains symptomatic with neuropathic medications and analgesics, optimization of diabetes management, and restoration of mobilization of the impaired muscle groups [16].

Diabetic amyotrophy is thought to have a favorable prognosis as it is a self-limiting condition. This demonstrates initial progressive worsening, which reaches a plateau after some time, followed by gradual recovery which can be either complete or with a residual motor deficit [7]. The disease progresses over months and can continue up to 2 years [20]. Progression to quadriparesis has been described in certain cases, albeit it is seldom characterized, with an unclear degree of recovery [21].

## Conclusion

This article describes the rare occurrence of radiculoplexus neuropathies, involving lumbar radiculoplexus, and the extremely uncommon involvement of cervical radiculoplexus as the only symptom which lead to an ultimate diagnosis of diabetes in two Sri Lankan males. Prior to the confirmation of DRPN, a comprehensive diagnostic workup is required to rule out other sinister causes, and a close follow-up for an adequate period of time is required to understand the evolution of symptoms, which may require reworkup with the possibility of revisiting the original diagnosis.

## Data Availability

Not applicable
